# Supplementary material for: Efficient and simultaneous capture of iodine and methyl iodide achieved by a covalent organic framework
Source: Nat Commun. 2022 May 24;13:2878. doi: 10.1038/s41467-022-30663-3 (PMC9130143; doi:10.1038/s41467-022-30663-3)
Supplement: Supplementary file 1 — Supplementary Information [file 41467_2022_30663_MOESM1_ESM.pdf]

Supplementary Information

Efficient and Simultaneous Capture of Iodine and Methyl Iodide Achieved by a Covalent Organic Framework

Xie et al.

Table of Contents

1. Experimental section······S3
2. Supplementary Tables 1-8······S7
3. Supplementary Figures 1-12······S15
4. References······S27

1. Experimental section

1.1 Materials and characterizations

All the starting materials and solvents were obtained from commercial sources and used without further purification. Anhydrous ethanol (EtOH, 99.9%), anhydrous *o*-dichlorobenzene (*o*-DCB, 99%), acetic acid (AcOH, 99%), anhydrous tetrahydrofuran (THF, 99.99%), anhydrous *N*,*N*–dimethylformamide (DMF, 99.99%), tris(4-formylphenyl)amine (TFPA, 97%), iodine (99.8%), and methyl iodide (99%) were purchased from Sigma−Aldrich. 1,3,5-tris(4-aminophenyl)benzene (TAPB, 97%) and 2,4,6-tri(4-aminophenyl)-1,3,5-triazine (TAPT, 97%) were purchased from TCI.

The Fourier transform infrared spectroscopy (FT-IR) spectra were collected using FT-IR spectrometer (Nicolet iS 10, Thermofisher Scientific). The solid-state ^{13}C Nuclear Magnetic Resonance (NMR) spectra were recorded on a Bruker Avance III WB-400 instrument with a Larmor frequency of 100.65 MHz. Elemental analysis of C, H, and N were carried out on an Elemental Vario EL III analyzer. The X-ray photoelectron spectroscopy (XPS) spectra were collected using a Kratos Supra spectrometer, equipped with a monochromatic Al Kα X-ray source (hν = 1,486.6 eV) at high vacuum using an aperture slot of 300 × 700 mm. High-resolution transmission electron microscopy (HRTEM) imaging was performed on an FEI Titan 80-300 microscope equipped with an image Cs corrector, operated at 300 kV. The powder X-ray diffraction (PXRD) patterns were recorded on Bruker D8 Advance instrument with Cu Kα radiation (λ = 0.1542 nm) under tube voltage and current of 40 kV and 40 mA, respectively. The nitrogen sorption isotherms at 77 K were collected by Micromeritics ASAP 2020. The Raman spectra were obtained using a LabRAM HR Evolution Raman spectrometer with a 532 nm laser. Inductively coupled plasma mass spectrometry (ICP-MS) measurements were carried out on an Elan DRC II spectrometer. The UV-vis diffuse reflectance measurement was performed on Cary 5000 UV-Vis-NIR spectrometer.

1.2 Preparation of the COF-TAPT and COF-TAPB

The COF-TAPT was prepared by mixing TFPA (1 mmol, 32.9 mg) and TAPT (1 mmol, 35.4 mg) in the presence of 6 M acetic acid (0.2 mL) with EtOH (0.4 mL) and *o*-DCB (1.6 mL) as solvent in a 10 mL Pyrex tube. After being sonicated for 10-15 minutes to form a homogenous dispersion, the mixture was flash frozen at 77 K and degassed by three freeze-pump-thaw cycles. Then, the tube was sealed and heated at 120 °C for 3 days. After the reaction, the product was purified by filtration and Soxhlet extraction in anhydrous THF for 48 hours, and dried under vacuum at 100 °C overnight. The synthesis of COF-TAPB follows a similar protocol by replacing TAPT with TAPB.

1.3 Preparation of the TFPA-TAPT

The control sample TFPA-TAPT was prepared by mixing TFPA (1 mmol, 32.9 mg) and TAPT (1 mmol, 35.4 mg) in the presence of 6 M acetic acid (0.2 mL) with DMF (2.0 mL) as solvent in a 10 mL Pyrex tube. After being sonicated for 10-15 minutes to form a homogenous dispersion, the mixture was flash frozen at 77 K and degassed by three freeze-pump-thaw cycles. Then, the tube was sealed off and heated at 120 °C for 3 days. After the reaction, the product was purified by filtration and Soxhlet extraction in anhydrous THF for 48 hours, and dried under vacuum at 100 °C overnight.

1.4 Structural simulation and PXRD analysis

For each material, an initial structure model was constructed based on the designed structure (1x1x1 supercell; eclipsed AA stacking mode) using Materials Studio suit of programs. The structure model was then geometrically optimized using Reflex modules. Starting from this initial unit cell, Pawley refinement was performed using TOPAS software to obtain a refined unit cell. During this process, the Pseudo-Voigt profile function was used for profile fitting, the fundamental instrumental parameters method was used for asymmetry correction, and line broadening related to crystallite size and lattice strain was considered. Lastly, the structure was further refined within the new unit cell to obtain the final atomic coordinates (see Supplementary Tables 1 and 2).

1.5 Static iodine and methyl iodide vapor capture

The static iodine (I_2) and methyl iodide (CH_3I) capture measurements were conducted in a customized system as illustrated in the scheme below. Typically, three 5 mL glass vials were placed in a 100 mL wide-mouth jar. The first vial was packed with the activated materials (COF-TAPT, COF-TAPB or TFPA-TAPT, 30.0 mg), the second one with I_2 or CH_3I, and the third one as a reference. Then the wide-mouth jar was sealed and kept at 75 °C in an oven. After a certain period of contact time, the wide-mouth jar was removed from the oven and cooled down to room temperature. The small glass vial containing adsorbents was weighted and placed back into the wide-mouth jar to continue the I_2 or CH_3I adsorption at 75 °C until its weight reached a steady value. The maximum static capture capacity was measured by the weight increment of the adsorbents. The static I_2 or CH_3I vapor capture uptake (q_t, g·g^{-1}) at certain time was calculated with Equation (S-1):

$$q_t = \frac{(m_t - m_1) - (M_t - M_0)}{(m_1 - m_0)} \qquad \text{Equation (S-1)}$$

where q_t (g·g^{-1}) denotes the static I_2 or CH_3I vapor capture uptake at time t, m_t (g) denotes the weight of the vial containing adsorbents at time t, m_1 (g) denotes the weight of the vial containing adsorbent before sorption, m_0 (g) denotes the weight of the empty vial for adsorbents, M_t (g) denotes the weight of the reference vial at time t, M_0 (g) denotes the weight of the reference vial before sorption.

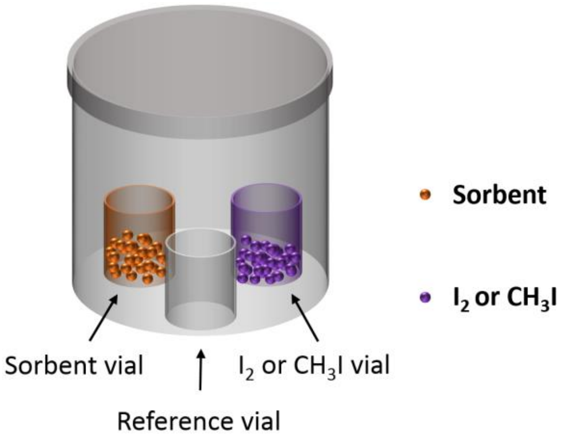

Scheme. Schematic illustration of the customized static I_2 or CH_3I vapor capture system.

1.6 Dynamic iodine and methyl iodide vapor capture

The I_2 capture breakthrough experiment was conducted using a lab-scale fixed-bed reactor at room temperature (25 °C) and 75 °C. In a typical experiment, 30.0 mg of the adsorbent was packed into a quartz column (5.8 mm inside diameter, 3 mm thickness, 150 mm length) with silane-treated glass wool filling the void space, and then activated at 150 °C under a helium flow (5 mL·min^{-1}) for 4 h. A dry nitrogen flow at a rate of 10 mL·min^{-1} was allowed to pass through an I_2 column and then the adsorbent column. The flow rate of I_2 was measured to be around 1.36 mg·hr^{-1}. The effluent from the adsorbent column was collected by 0.3 M NaOH aqueous solution for scrubbing halogen gases including I_2 and HI, if present. The I content in the NaOH aqueous solution was detected by ICP-MS. Experiments in the presence of humidity were performed by injecting water into the gas mixture at a rate of 0.32 mL·min^{-1} using a Fusion 100 syringe pump. For modulating 150 ppm I_2, a dry nitrogen flow at a rate of 150 mL·min^{-1} was allowed to pass through an I_2 column and then mixed with a dilution nitrogen flow at a rate of 250 mL·min^{-1} before passing through the adsorbent column. The flow rate of I_2 was measured to be around 13.004 mg·hr^{-1}.

For the breakthrough measurement for CH_3I capture, the adsorbent was activated following the same procedure described above. A dry nitrogen flow at a rate of 3 mL·min^{-1} was bubbled through CH_3I column and then passed through the adsorbent column. The flow rate of CH_3I was measured to be around 4.408 mg·min^{-1}. The effluent from the adsorbent column was monitored using an online MS. Experiments in the presence of humidity were performed by injecting water into the gas mixture using a Fusion 100 syringe pump. The absolute adsorbed amount of gas i (q_i) was calculated from the breakthrough curve using Equation (S-2):

$$q_{\mathrm{i}} = \frac{F_{\mathrm{i}} \times t_0 - \mathrm{V}_{\mathrm{dead}} - \int_0^{t_0} F_{\mathrm{e}} \Delta \mathrm{t}}{m} \qquad \text{Equation (S-2)}$$

where F_i denotes the influent flow rate of the specific gas (mL·min^{-1}); t_0 denotes the adsorption time (min); V_{dead} denotes the dead volume of the system (cm^3); F_e denotes the effluent flow rate of the specific gas (mL·min^{-1}); and m denotes the weight of the adsorbents (g).

For modulating 50 ppm CH_3I, a dry nitrogen flow with a rate of 0.1 mL·min^{-1} was allowed to pass through the CH_3I column and then mixed with a dilution nitrogen flow at a rate of 400 mL·min^{-1} before passing through the adsorbent column. The flow rate of CH_3I was measured to be around 4.332 mg·hr^{-1}. Given that 50 ppm CH_3I is out of the detection limit of MS, the CH_3I uptake at this concentration was calculated based on the weight increment after achieving adsorption saturation.

To compose a simulating off-gas with 150 ppm of I_2 and 50 ppm of CH_3I, a dry nitrogen flow at a rate of 150 mL·min^{-1} was allowed to pass through an I_2 column, while another dry nitrogen flow at a rate of 0.1 mL·min^{-1} was allowed to pass through CH_3I column. Subsequently, these two flows were combined and then mixed with a dilution nitrogen flow at a rate of 250 mL·min^{-1} before passing through the adsorbent column. The flow rates of I_2 and CH_3I were measured to be around 13.004 and 4.332 mg·hr^{-1}, respectively.

1.7 Material regeneration

Given that the unprecedented static and dynamic I_2 and CH_3I vapor capture capacities make COF-TAPT a promising adsorbent for I_2 and CH_3I, its regeneration was further explored. A solvent treatment was performed to release the adsorbed I_2/CH_3I and regenerate the adsorbent. Typically, the I_2-saturated COF-TAPT was immersed into ethanol or acetone, and ultrasonicated for a certain period of time. The I_2 desorption process was monitored by measuring the concentration of I_2 in ethanol with ultraviolet–visible spectroscopy (UV–Vis). The CH_3I-saturated COF-TAPT can be regenerated following a similar

protocol. The regenerated samples were dried under vacuum at 100 °C overnight and then submitted for the next round of dynamic I_2/CH_3I vapor capture experiments at 25 °C.

2. Supplementary Tables 1-8

Supplementary Table 1 Fractional atomic coordinates in the refined unit cell of COF-TAPT.

COF-TAPT: Space group: P3							
a=23.51 Å, *b*=23.51 Å, *c*=3.64 Å							
α=90°, *β*=90°, *γ*=120°							
Atom	x	y	z	Atom	x	y	z
C1	-0.37444	-0.63645	0.55262	C14	-0.48428	-0.38996	0.41064
C2	-0.23123	-0.56122	0.69341	C15	-0.62997	-0.36343	0.44952
C3	-0.16293	-0.52157	0.69101	N16	-0.69661	-0.39978	0.44962
C4	-0.12379	-0.54581	0.55312	H17	-0.26303	-0.54158	0.81081
C5	-0.15349	-0.60986	0.41425	H18	-0.13906	-0.46927	0.80193
C6	-0.22183	-0.64899	0.41171	H19	-0.12183	-0.63013	0.30299
C7	-0.49657	-0.54881	0.55602	H20	-0.24594	-0.70085	0.29414
N8	-0.47420	-0.48786	0.46745	H21	-0.55031	-0.58054	0.63806
C9	-0.51422	-0.45782	0.46298	H22	-0.60746	-0.55023	0.55099
C10	-0.58264	-0.49485	0.50874	H23	-0.67590	-0.49437	0.53877
C11	-0.62035	-0.46408	0.50266	H24	-0.49712	-0.30374	0.36542
C12	-0.59028	-0.39597	0.45230	H25	-0.42881	-0.35964	0.37122
C13	-0.52199	-0.35916	0.40641	N26	-0.33333	-0.66667	0.55264

Supplementary Table 2 Fractional atomic coordinates in the refined unit cell of COF-TAPB.

COF-TAPB: Space group: P3							
a=23.61 Å, *b*=23.61 Å, *c*=3.65 Å							
α=90°, *β*=90°, *γ*=120°							
Atom	x	y	z	Atom	x	y	z
C1	-0.37424	-0.63656	0.55408	C15	-0.62889	-0.36439	0.44861
C2	-0.23166	-0.56175	0.69540	C16	-0.69738	-0.40139	0.44870
C3	-0.16365	-0.52230	0.69305	H17	-0.26330	-0.54222	0.81319
C4	-0.12470	-0.54641	0.55453	H18	-0.13986	-0.47027	0.80451
C5	-0.15430	-0.61013	0.41510	H19	-0.12280	-0.63029	0.30331
C6	-0.22235	-0.64908	0.41263	H20	-0.24638	-0.70068	0.29469
C7	-0.49577	-0.54922	0.55749	H21	-0.54914	-0.58065	0.64244
N8	-0.47363	-0.48870	0.46575	H22	-0.60598	-0.55036	0.56298
C9	-0.51344	-0.45875	0.46157	H23	-0.67406	-0.49467	0.55137
C10	-0.58138	-0.49537	0.51482	H24	-0.49658	-0.30568	0.35076
C11	-0.61888	-0.46470	0.50902	H25	-0.42869	-0.36140	0.35660
C12	-0.58923	-0.39700	0.45130	H26	-0.72266	-0.45707	0.44884
C13	-0.52129	-0.36070	0.39789	N27	-0.33333	-0.66667	0.55411
C14	-0.48378	-0.39140	0.40202				

Supplementary Table 3 Textural properties, static I_2 uptakes, and static CH_3I uptakes of our COF-TAPT and COF-TAPB, the control sample TFPA-TAPT, and four previously reported MOF- and COF-based adsorbents (MIL-101-Cr-HMTA, SCU-COF-2, TPB-DMTP-COF, and COF-OH-0).

Sample	S_{BET} ($m^2 \cdot g^{-1}$) [a]	Pore volume ($cm^3 \cdot g^{-1}$)	N content ($mmol \cdot g^{-1}$)	Static I_2 uptake ($g \cdot g^{-1}$)[b]	Static CH_3I uptake ($g \cdot g^{-1}$)[c]
COF-TAPT	2348	0.97	11.12	8.61	1.53
TFPA-TAPT	1284	0.19	11.12	4.31	1.37
COF-TAPB	2290	0.89	6.39	7.94	0.81
MIL-101-Cr-HMTA[d]	2272	--	8.68	5.17	1.35
SCU-COF-2[e]	413.4	--	10.75	6.0	1.45
TPB-DMTP-COF[f]	1766	1.30	5.10	5.85	0.70
COF-OH-0 [g]	2209	1.83	10.15	6.31	1.40

[a]:BET surface area.

[b]: Measured at 75 °C.

[c]: Measured at 75 °C.

[d]: This material was provided by Baiyan Li et al.[2]

[e]: Data is from ref[3].

[f]: This material was prepared based on the method reported in ref[4].

[g]: This material was prepared based on the method reported in ref[1].

Supplementary Table 4 Elemental analysis results of COF-TAPT, COF-TAPB, and TFPA-TAPT.

Sample	Theoretical content			Experimental content		
	C	N	H	C	N	H
COF-TAPT	80.09%	15.57%	4.34%	79.73%	14.87%	5.4%
COF-TAPB	86.22%	8.94%	4.85%	85.87%	8.21%	5.92%
TFPA-TAPT	80.09%	15.57%	4.34%	79.54%	14.7%	5.59%

Supplementary Table 5 Static I_2 vapor capture performances of various adsorbents.

Type	Adsorbent	T (°C)	S_{BET} ($m^2 \cdot g^{-1}$)	Pore volume ($cm^3 \cdot g^{-1}$)	I_2 uptake ($g \cdot g^{-1}$)	$t_{80\%}$ (h)[b]	$K_{80\%}$ ($g \cdot g^{-1} \cdot h^{-1}$)[c]	Ref.
POPs	BisImi-POP@2	77	--	--	10.30	21	0.49	[5]
	TBIM	77	8.12	0.02	9.43	23	0.41	[6]
	60PEI@HCP	75	538	0.45	6.07	3	2.02	[7]
	PSIF-5a	75	574	1.41	5.75	5	1.15	[8]
	CMP-LS8	80	2028	2.07	5.29	1.5	3.53	[9]
	HCP-V2	75	750	--	5.25	30	0.18	[10]
	CMPN	75	86.2	0.218	5.03	1	5.03	[11]
	CSU-CPOPs-1	75	1032	1.11	4.95	8	0.62	[12]
	TTPA	77	308	0.36	4.92	12	0.41	[13]
	TTPPA	77	512.39	0.30	4.90	18	0.27	[14]
	TatPOP-2	75	36.5	0.22	4.5	1	4.50	[15]
	TTPB	77	222	0.3	4.43	11	0.40	[16]
	CMP-LS5	80	1185	1.36	4.4	1.8	2.44	[17]
	PG-800	80	1755	1.31	4.11	0.67	6.13	[18]
	PHF-1-Ct	80	690	0.44	4.05	13	0.31	[19]
	PHF-1	80	1046	0.61	3.05	8	0.38	[19]
	CTF-CTTD-500	75	1334	1.40	3.87	21	0.18	[20]
	HCMP-3	75	92	0.6	3.36	22	0.15	[21]
	PTPATTh	70	594	1.469	3.13	18	0.17	[22]
	CalP4-Li	75	445	0.588	3.12	1	3.12	[23]
	CalP4	75	759	1.08	2.2	5.3	0.42	[23]
	AzoPPN	77	400	0.86	2.9	11	0.26	[24]
	PAF-24	75	136	--	2.76	--	--	[25]
	BDP-CPP-1	75	635	0.78	2.83	5	0.57	[26]
	CTF-Cl-4	75	889	0.58	3.12	5	0.60	[27]
MOFs	IL@PCN-333(Al)	75	1635.3	1.40	7.35	3	2.45	[28]
	PCN-333(Al)	75	2935.9	2.97	4.42	--	--	[28]
	HKUST-1@PES	75	376	--	5.38	18	0.30	[29]
	MOF-808	80	1930	0.82	2.18	13	0.17	[30]
Porous carbon	SR-KOH	77	3072	1.77	6.46	1	6.46	[31]
H_cOFs	H_cOFs	75	--	--	4.73	--	--	[32]
COFs	iCOF-AB-50	75	1390	1.21	10.21	7.25	1.12	[1]
	QTD-COF-V	75	--	--	6.29	2.5	2.52	[33]
	TPB-DMTP	77	1927	1.28	6.20	36	0.17	[4]
	SCU-COF-2	75	413.4	0.46	6.0	24	0.25	[3]
	TJNU-201	77	2510	--	5.625	18	0.31	[34]
	TPT-BD-COF	75	109	0.3	5.43	8	0.68	[35]
	SIOC-COF-7	75	618	0.41	4.81	15	0.32	[36]
	COF-DL229	75	1762	0.64	4.7	--	--	[37]
	COF-TAPT	75	2348	0.97	8.61	18	0.48	This work
	COF-TAPB	75	2290	0.89	7.94	24	0.33	

[a] Due to the lack of direct data, we estimated the $t_{80\%}$ using the time-dependent adsorption curves at their initial stages.
[b] Estimated $K_{80\%}$.

Supplementary Table 6 Dynamic I_2 vapor capture performances of various adsorbents. The I_2 concentration is 400 ppm unless stated otherwise.

Type	Adsorbent	Temperature	Pressure	I_2 uptake ($g \cdot g^{-1}$)	Ref.
Zeolite	HISL	R.T.	Ambient	0.53	[38]
	SL-1	R.T.	Ambient	0.48	[38]
	Si-BEA	R.T.	Ambient	0.47	[38]
	Ag^0@MOR	R.T.	Ambient	0.08	[2]
Inorganic material	AC	R.T.	Ambient	0.70	[38]
	Zn-Sacc	R.T.	Ambient	0.05	[38]
MOFs	ZIF-8	R.T.	Ambient	0.03	[38]
	HKUST-1	R.T.	Ambient	0.38	[38]
	MIL-101-TED	423 K	Ambient	0.43[a]	[2]
	MIL-101-Cr-HMTA	423 K	Ambient	0.41[a]	[2]
		R.T.	Ambient	0.84[b]	--
COFs	iCOF-AB-50	25	Ambient	2.79	[1]
	SCU-COF-2	R.T.	Ambient	0.98	[3]
	COF-TAPT	25	Ambient	2.38	This work
	COF-TAPB	25	Ambient	2.18	

[a] The measurements were conducted at an I_2 concentration of 150 ppm.

[b] The measurement was conducted in this work.

Supplementary Table 7 Dynamic CH_3I vapor capture performances of various adsorbents at a CH_3I concentration of 200,000 ppm.

Type	Adsorbent	Temperature (°C)	Pressure	CH_3I uptake ($g{\cdot}g^{-1}$)	Ref.
Zeolite	Ag^{+}@13X	30	Ambient	0.45 [a]	2
	HISL	30	Ambient	0.42 [a]	38
	Ag^{+}@MOR	30	Ambient	0.29 [a]	2
	Ag^{+}@ZSM5	30	Ambient	0.28 [a]	2
	Ag^{0}@MOR	30	Ambient	0.25 [a]	2
Composites	HMTA@AC	30	Ambient	0.54 [a]	2
	TED@AC	30	Ambient	0.52 [a]	2
MOFs	MIL-101-Cr-HMTA	25	Ambient	1.66 [b]	
		30	Ambient	1.74 [a]	2
	MIL-101-Cr-TED	30	Ambient	1.60 [a]	2
COFs	iCOF-AB-50	25	Ambient	0.62 [b]	1
	SCU-COF-2	25	Ambient	0.564 [b]	3
	COF-TAPT	25	Ambient	1.30 [b]	This work
	COF-TAPB	25	Ambient	0.71 [b]	

[a] The uptakes were obtained based on thermogravimetric analysis.

[b] The uptakes were obtained based on breakthrough curves in this work.

Supplementary Table 8 Dynamic capture performances of various adsorbents at 150 ppm I_2 vapor, 50 ppm CH_3I vapor, and the mixture vapor of 150 ppm I_2 and 50 ppm CH_3I.

Sample	Dynamic uptake ($g \cdot g^{-1}$) at 25 °C		
	150 ppm I_2	50 ppm CH_3I	150 ppm I_2 + 50 ppm CH_3I
COF-TAPT	1.25	0.39	1.51
COF-TAPB	1.12	0.12	1.17
TFPA-TAPT	0.42	0.18	0.47
MIL-101-Cr-HMTA[2]	0.83	0.51	1.08
SCU-COF-2[3]	0.49	0.08	0.56
iCOF-AB-50[1]	1.52	0.11	1.59

3. Supplementary Figures 1-12

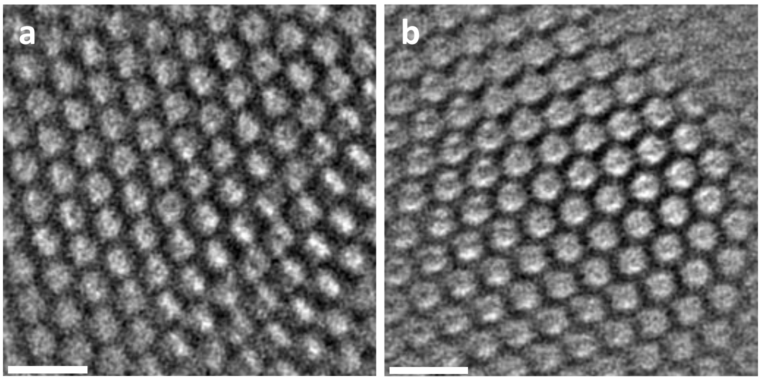

Supplementary Fig. 1 HRTEM images (denoised via an average background subtraction filter) of (**a**) COF-TAPT and (**b**) COF-TAPB. Scale bar: 5 nm.

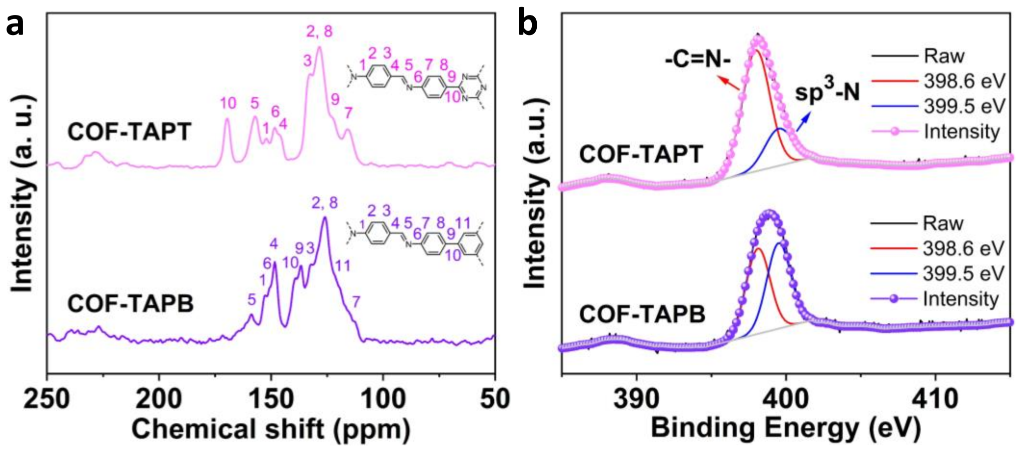

Supplementary Fig. 2 (**a**) N 1*s* XPS spectra of COF-TAPT and COF-TAPB. (**b**) Solid state ^{13}C NMR spectra of COF-TAPT and COF-TAPB.

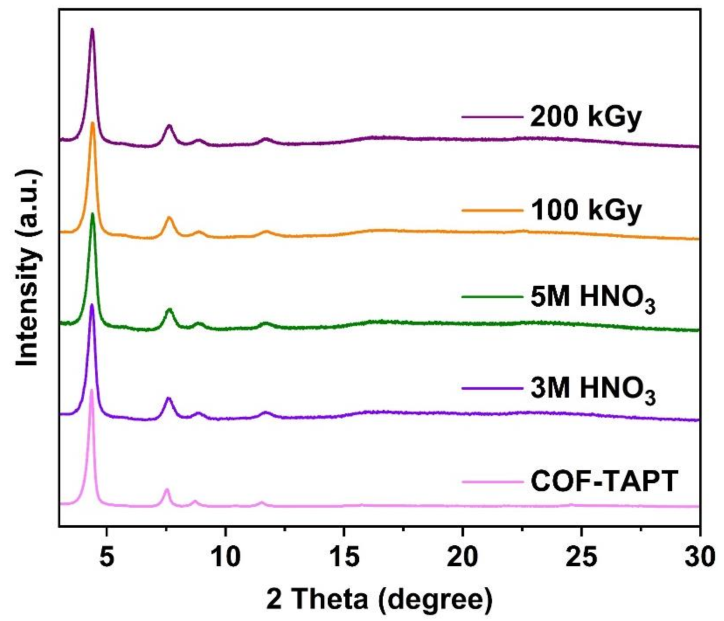

Supplementary Fig. 3 PXRD patterns of COF-TAPT before and after various treatments, including acid treatments with concentrated HNO_3 aqueous solution (3M, 5M) for 48 h, and irradiation treatments with β-irradiation (100 kGy, 200 kGy) provided by an electron accelerator (1.0 MeV, Wasik Associates Inc., USA).

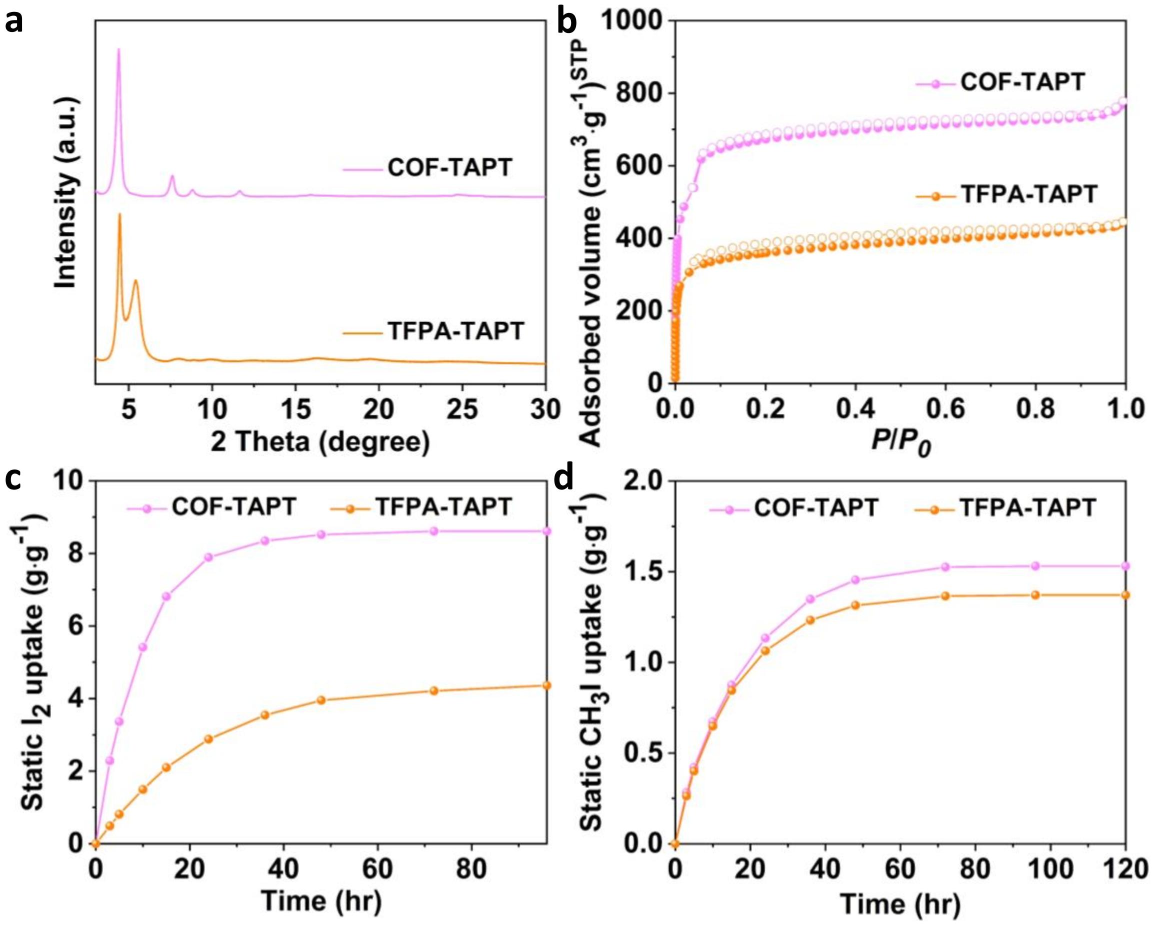

Supplementary Fig. 4 (**a**) PXRD patterns, (**b**) N_2 sorption isotherms, (**c**) time-dependent static I_2 vapor adsorption curves at 75 °C and (**d**) time-dependent static CH_3I vapor adsorption curves at 75 °C of COF-TAPT and TFPA-TAPT.

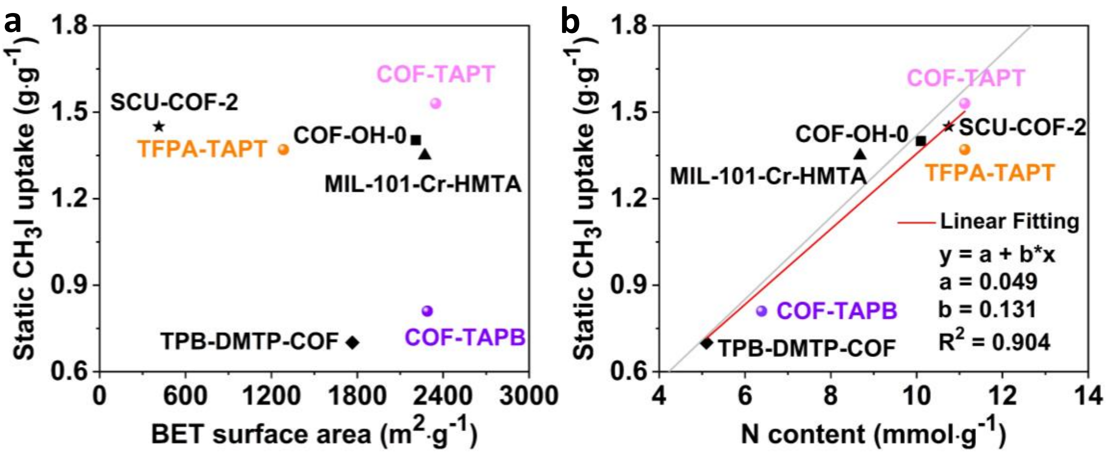

Supplementary Fig. 5 Relationships between static CH_3I uptakes at 75 °C of different samples and their (a) BET surface areas, and (b) N contents. The grey line in (**b**) represents the relationship between theoretical CH_3I uptake and N content assuming that each N atom interacts with one CH_3I molecule. The red line in (**b**) is the linear fitting result based on the seven data points, giving a reasonably good coefficient of determination (R^2) of 0.904.

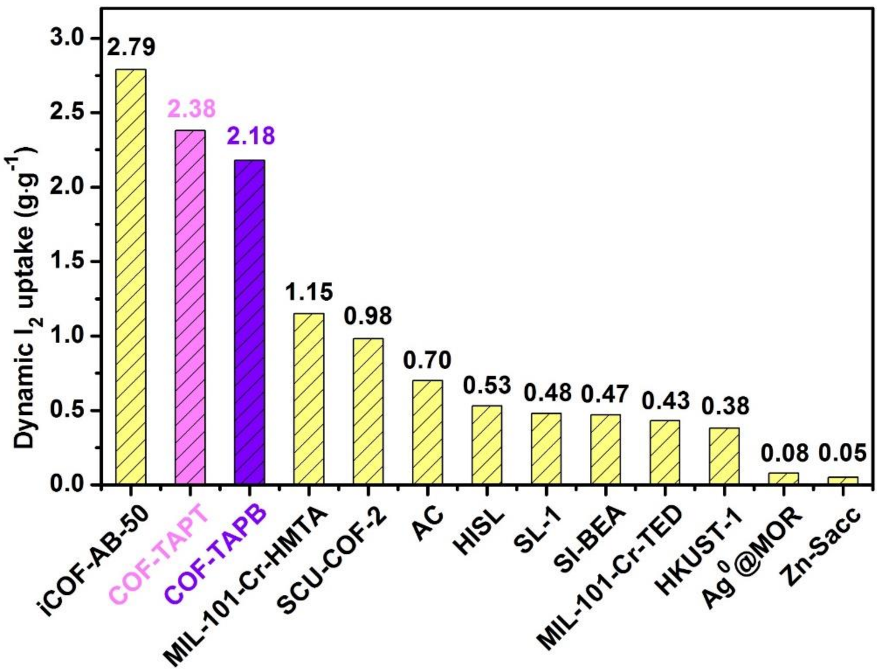

Supplementary Fig. 6 Comparison of dynamic I_2 uptake of prepared COF materials with the representative adsorbents for I_2 capture at 25 °C with 400 ppm I_2.

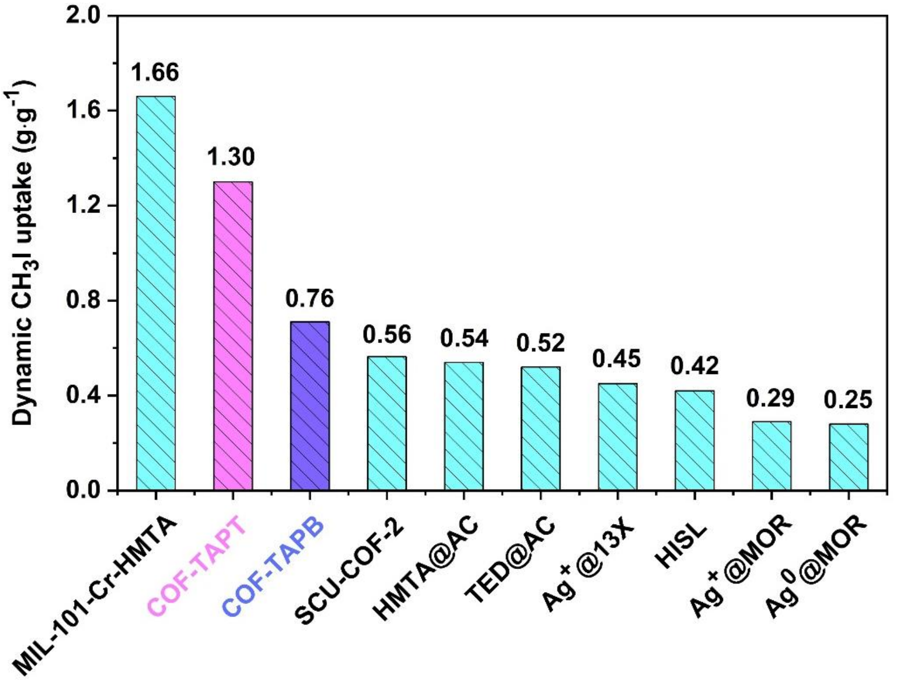

Supplementary Fig. 7 Comparison of dynamic CH_3I uptake of prepared COF materials with the representative adsorbents for CH_3I capture at 25 °C with 200,000 ppm CH_3I.

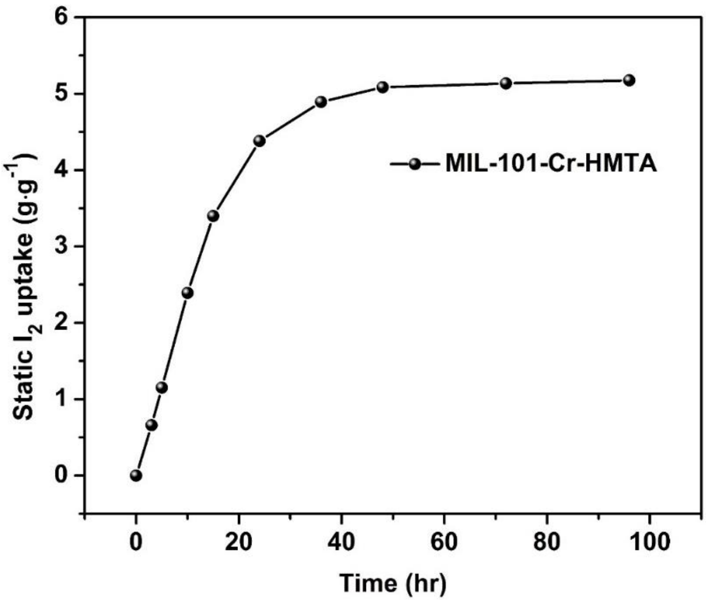

Supplementary Fig. 8 Gravimetric measurement of static I_2 vapor adsorption capacities of MIL-101-Cr-HMTA adsorbent as a function of time at 75 °C. Sample was provided by Baiyan Li et al.[2]

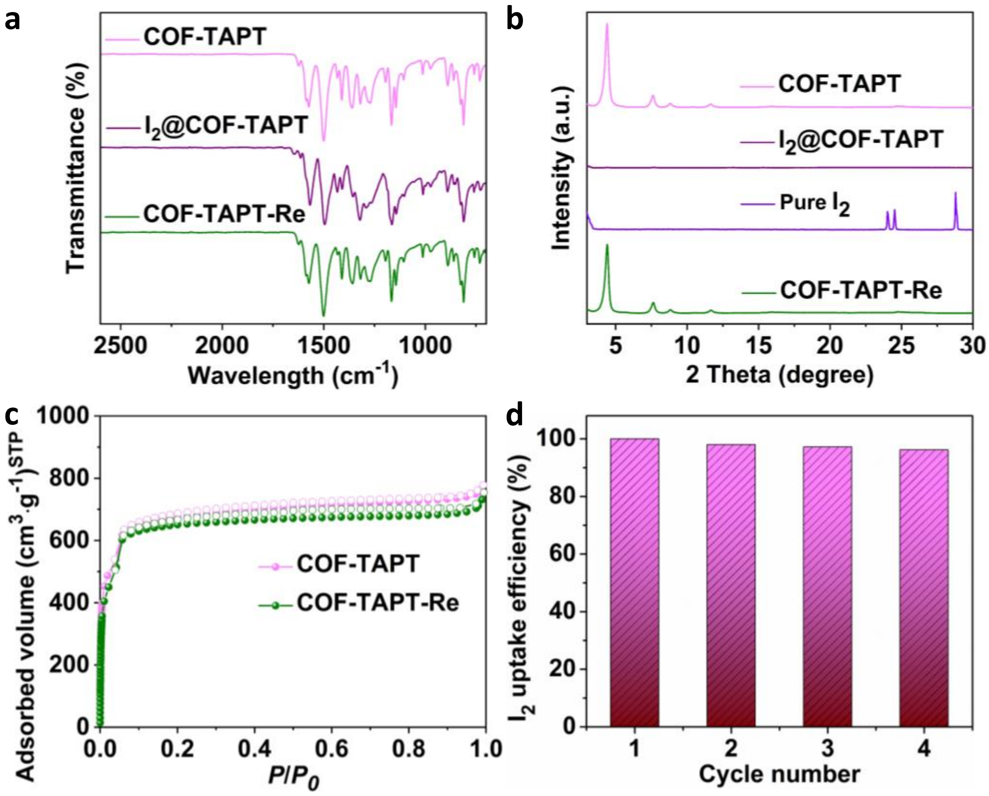

Supplementary Fig. 9 Structural characterizations and regeneration ability of COF-TAPT during I_2 adsorption. (**a**) FT-IR spectra of COF-TAPT, I_2@COF-TAPT, and COF-TAPT-Re; (**b**) PXRD patterns of COF-TAPT, I_2@COF-TAPT, pure I_2, and COF-TAPT-Re; (**c**) N_2 sorption of COF-TAPT and COF-TAPT-Re; (**d**) Dynamic I_2 adsorption capacity of COF-TAPT in four successive adsorption/extraction cycles at 25 °C with 400 ppm I_2. "I_2@COF-TAPT" refers to I_2-satuarated COF-TAPT. "COF-TAPT-Re" refers to regenerated COF-TAPT after one adsorption/extraction cycle.

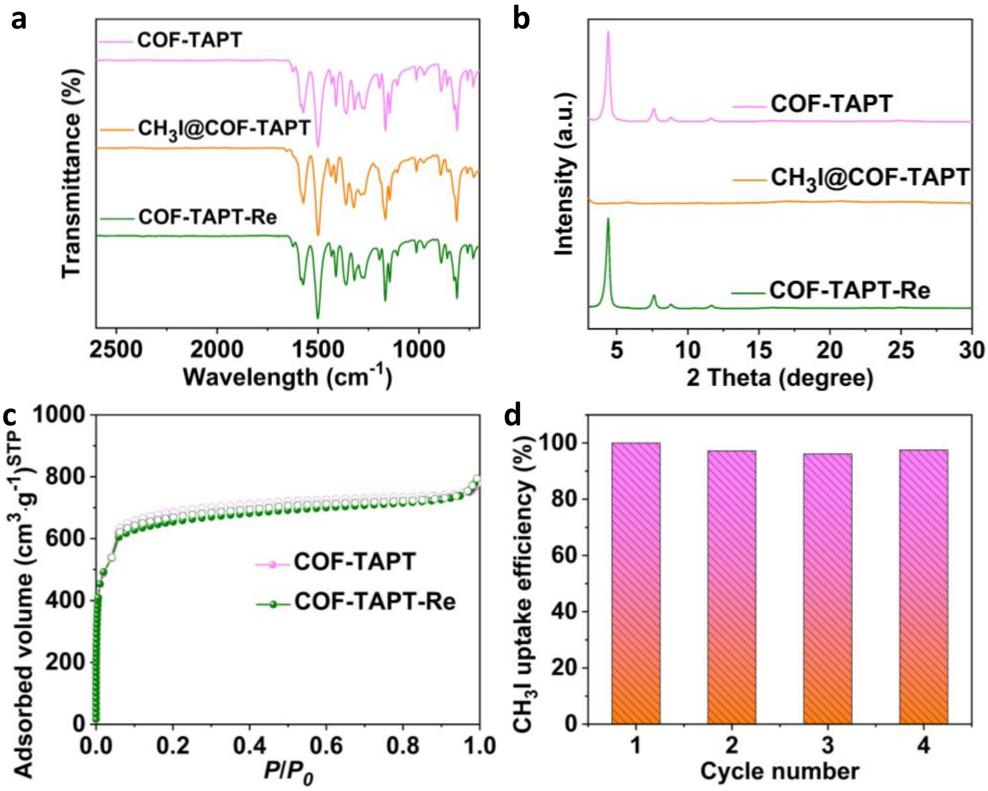

Supplementary Fig. 10 Structural characterizations and regeneration ability of COF-TAPT during CH_3I adsorption. (**a**) FT-IR spectra of COF-TAPT, CH_3I@COF-TAPT, and COF-TAPT-Re; (**b**) PXRD patterns of COF-TAPT, CH_3I@COF-TAPT, and COF-TAPT-Re; (**c**) N_2 sorption of COF-TAPT and COF-TAPT-Re; (**d**) Dynamic CH_3I adsorption capacity of COF-TAPT in four successive adsorption/extraction cycles at 25 °C with 200,000 ppm CH_3I. "CH_3I@COF-TAPT" refers to CH_3I-satuarated COF-TAPT. "COF-TAPT-Re" refers to regenerated COF-TAPT after one adsorption/extraction cycle toward CH_3I.

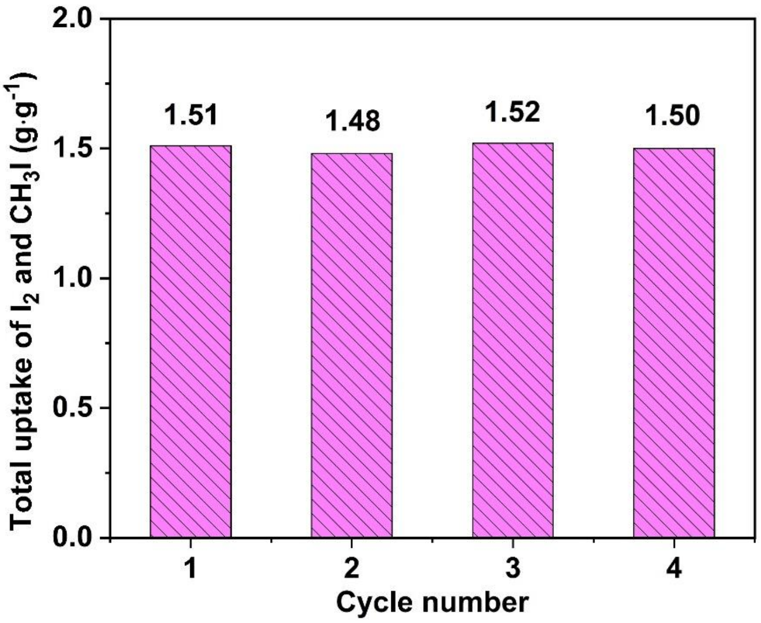

Supplementary Fig. 11 Dynamic adsorption capacity of COF-TAPT with a stream of 150 ppm I_2 and 50 ppm CH_3I in four successive adsorption/extraction cycles at 25 °C.

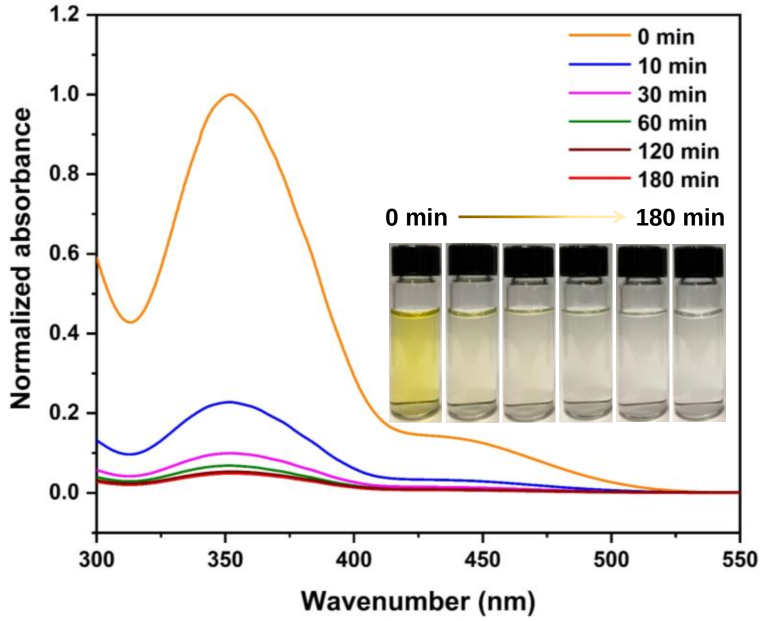

Supplementary Fig. 12 UV-vis absorption spectra of 0.1 M $K_2Cr_2O_7$ aqueous solution collected at specific times during the anion exchange process. The CH_3I saturated COF-TAPT (CH_3I@COF-TAPT) was used as anion exchanger. The insets are photo pictures, showing the apparent discoloration of the solution during the process. The experiment was conducted as follows: 50 mg of CH3I@COF-TAPT was added to a 200 mL beaker containing 100 mL of potassium dichromate ($K_2Cr_2O_7$, 0.1 mM) solution. The mixture was kept stirring at 25 °C; at specific time points, 2 mL of sample was taken from the mixture, separated with a 0.22 mm nylon membrane filter, and measured by UV-vis spectroscopy.

4. References

1. Xie, Y., et al. Ionic Functionalization of Multivariate Covalent Organic Frameworks to Achieve an Exceptionally High Iodine-Capture Capacity. *Angewandte Chemie International Edition* **60**, 22432–22440 (2021).
2. Li, B., et al. Capture of organic iodides from nuclear waste by metal-organic framework-based molecular traps. *Nature Communications* **8**, 485 (2017).
3. He, L., et al. A nitrogen-rich covalent organic framework for simultaneous dynamic capture of iodine and methyl iodide. *Chem* **7**, 699–714 (2021).
4. Wang, P., Xu, Q., Li, Z., Jiang, W., Jiang, Q., Jiang, D. Exceptional iodine capture in 2D covalent organic frameworks. *Advanced Materials* **30**, 1801991 (2018).
5. Niu, T.-H., Feng, C.-C., Yao, C., Yang, W.-Y., Xu, Y.-H. Bisimidazole-Based Conjugated Polymers for Excellent Iodine Capture. *ACS Applied Polymer Materials* **3**, 354–361 (2020).
6. Geng, T., Zhang, C., Liu, M., Hu, C., Chen, G. Preparation of biimidazole-based porous organic polymers for ultrahigh iodine capture and formation of liquid complexes with iodide/polyiodide ions. *Journal of Materials Chemistry A* **8**, 2820–2826 (2020).
7. Li, X., Chen, G., Jia, Q. Highly efficient iodine capture by task-specific polyethylenimine impregnated hypercrosslinked polymers. *Journal of the Taiwan Institute of Chemical Engineers* **93**, 660–666 (2018).
8. Janeta, M., Bury, W., Szafert, S. Porous silsesquioxane–imine frameworks as highly efficient adsorbents for cooperative iodine capture. *ACS Applied Materials & Interfaces* **10**, 19964–19973 (2018).
9. Wang, S., et al. Multifunctional conjugated microporous polymers with pyridine unit for efficient iodine sequestration, exceptional tetracycline sensing and removal. *Journal of Hazardous Materials* **387**, 121949–121958 (2020).
10. Li, X., Chen, G., Jia, Q. One-pot synthesis of viologen-based hypercrosslinked polymers for efficient volatile iodine capture. *Microporous and Mesoporous Materials* **279**, 186–192 (2019).
11. Xu, M., Wang, T., Zhou, L., Hua, D. Fluorescent conjugated mesoporous polymers with N, N-diethylpropylamine for the efficient capture and real-time detection of volatile iodine. *Journal of Materials Chemistry A* **8**, 1966–1974 (2020).
12. Xiong, S., Tang, X., Pan, C., Li, L., Tang, J., Yu, G. Carbazole-bearing porous organic polymers with a mulberry-like morphology for efficient iodine capture. *ACS Applied Materials & Interfaces* **11**, 27335–27342 (2019).
13. Geng, T., Zhang, W., Zhu, Z., Kai, X. Triazine-based conjugated microporous polymers constructing triphenylamine and its derivatives with nitrogen as core for iodine adsorption and fluorescence sensing I_2. *Microporous and Mesoporous Materials* **273**, 163–170 (2019).
14. Geng, T., Ye, S., Zhu, Z., Zhang, W. Triazine-based conjugated microporous polymers with N, N, N', N'-tetraphenyl-1, 4-phenylenediamine, 1, 3, 5-tris (diphenylamino) benzene and 1, 3, 5-tris [(3-methylphenyl)-phenylamino] benzene as the core for high iodine capture and fluorescence sensing of o-nitrophenol. *Journal of Materials Chemistry A* **6**, 2808–2816 (2018).
15. Xiong, S., et al. Uniform poly (phosphazene–triazine) porous microspheres for highly efficient iodine removal. *Chemical Communications* **54**, 8450–8453 (2018).

16. Geng, T., Zhu, Z., Zhang, W., Wang, Y. A nitrogen-rich fluorescent conjugated microporous polymer with triazine and triphenylamine units for high iodine capture and nitro aromatic compound detection. *Journal of Materials Chemistry A* **5**, 7612–7617 (2017).

17. Wang, S., et al. Ultrahigh volatile iodine capture by conjugated microporous polymer based on N, N, N', N'-tetraphenyl-1, 4-phenylenediamine. *Polymer Chemistry* **10**, 2608–2615 (2019).

18. Sun, H., et al. Efficient capture and reversible storage of radioactive iodine by porous graphene with high uptake. *ChemistrySelect* **3**, 10147–10152 (2018).

19. Jie, K., et al. A benzoquinone-derived porous hydrophenazine framework for efficient and reversible iodine capture. *Chemical Communications* **54**, 12706–12709 (2018).

20. Jiang, Q., Huang, H., Tang, Y., Zhang, Y., Zhong, C. Highly porous covalent triazine frameworks for reversible iodine capture and efficient removal of dye. *Industrial & Engineering Chemistry Research* **57**, 15114–15121 (2018).

21. Liao, Y., Weber, J., Mills, B. M., Ren, Z., Faul, C. F. Highly efficient and reversible iodine capture in hexaphenylbenzene-based conjugated microporous polymers. *Macromolecules* **49**, 6322–6333 (2016).

22. Geng, T., Chen, G., Xia, H., Zhang, W., Zhu, Z., Cheng, B. Poly {tris [4-(2-thienyl) phenyl] amine} and poly [tris (4-carbazoyl-9-yl phenyl) amine] conjugated microporous polymers as absorbents for highly efficient iodine adsorption. *Journal of Solid State Chemistry* **265**, 85–91 (2018).

23. Shetty, D., Raya, J., Han, D. S., Asfari, Z., Olsen, J.-C., Trabolsi, A. Lithiated polycalix[4]arenes for efficient adsorption of iodine from solution and vapor phases. *Chemistry of Materials* **29**, 8968–8972 (2017).

24. Li, H., Ding, X., Han, B. H. Porous azo-bridged porphyrin-phthalocyanine network with high iodine capture capability. *Chemistry–A European Journal* **22**, 11863–11868 (2016).

25. Yan, Z., Yuan, Y., Tian, Y., Zhang, D., Zhu, G. Highly efficient enrichment of volatile iodine by charged porous aromatic frameworks with three sorption sites. *Angewandte Chemie International Edition* **54**, 12733–12737 (2015).

26. Zhu, Y., et al. BODIPY-based conjugated porous polymers for highly efficient volatile iodine capture. *Journal of Materials Chemistry A* **5**, 6622–6629 (2017).

27. Xu, G., Zhu, Y., Xie, W., Zhang, S., Yao, C., Xu, Y. Porous cationic covalent triazine-based frameworks as platforms for efficient CO2 and iodine capture. *Chemistry–An Asian Journal* **14**, 3259–3263 (2019).

28. Tang, Y., Huang, H., Li, J., Xue, W., Zhong, C. IL-induced formation of dynamic complex iodide anions in IL@MOF composites for efficient iodine capture. *Journal of Materials Chemistry A* **7**, 18324–18329 (2019).

29. Bardiya, V., Tu, N. N., Berend, S., Kyriakos, C. S. Porous Metal–Organic Framework@Polymer Beads for Iodine Capture and Recovery Using a Gas-Sparged Column. *Advanced Functional Materials* **28**, 1801596 (2018).

30. Chen, P., He, X., Pang, M., Dong, X., Zhao, S., Zhang, W. Iodine Capture Using Zr-Based Metal–Organic Frameworks (Zr-MOFs): Adsorption Performance and Mechanism. *ACS Applied Materials & Interfaces* **12**, 20429–20439 (2020).

31. Sun, H., Yang, B., Li, A. Biomass derived porous carbon for efficient capture of carbon dioxide, organic contaminants and volatile iodine with exceptionally high uptake. *Chemical Engineering Journal* **372**, 65–73 (2019).

32. Jiang, X., et al. Topochemical Synthesis of Single-Crystalline Hydrogen-Bonded Cross-Linked Organic Frameworks and Their Guest-Induced Elastic Expansion. *Journal of the American Chemical Society* **141**, 10915–10923 (2019).

33. Guo, X., et al. Colyliform Crystalline 2D Covalent Organic Frameworks with Quasi-3D Topologies for Rapid I_2 Adsorption. *Angewandte Chemie* **132**, 22886–22894 (2020).

34. Li, J., et al. Two-dimensional covalent–organic frameworks for ultrahigh iodine capture. *Journal of Materials Chemistry A* **8**, 9523–9527 (2020).

35. Guo, X., et al. Mechanistic insight into hydrogen-bond-controlled crystallinity and adsorption property of covalent organic frameworks from flexible building blocks. *Chemistry of Materials* **30**, 2299–2308 (2018).

36. Yin, Z.-J., Xu, S.-Q., Zhan, T.-G., Qi, Q.-Y., Wu, Z.-Q., Zhao, X. Ultrahigh volatile iodine uptake by hollow microspheres formed from a heteropore covalent organic framework. *Chemical Communications* **53**, 7266–7269 (2017).

37. Wang, C., et al. A 3D covalent organic framework with exceptionally high iodine capture capability. *Chemistry–A European Journal* **24**, 585–589 (2018).

38. Pham, T. C. T., et al. Capture of iodine and organic iodides using silica zeolites and the semiconductor behaviour of iodine in a silica zeolite. *Energy & Environmental Science* **9**, 1050–1062 (2016).
